# Toll-like receptors in the pathogenesis of human B cell malignancies

**DOI:** 10.1186/s13045-014-0057-5

**Published:** 2014-08-12

**Authors:** Johana M Isaza-Correa, Zheng Liang, Anke van den Berg, Arjan Diepstra, Lydia Visser

**Affiliations:** 1 Department of Pathology and Medical Biology, University of Groningen, University Medical Center Groningen, Hanzeplein 1 HPC EA10, Groningen 9700RB, The Netherlands

## Abstract

Toll-like receptors (TLRs) are important players in B-cell activation, maturation and memory and may be involved in the pathogenesis of B-cell lymphomas. Accumulating studies show differential expression in this heterogeneous group of cancers. Stimulation with TLR specific ligands, or agonists of their ligands, leads to aberrant responses in the malignant B-cells. According to current data, TLRs can be implicated in malignant transformation, tumor progression and immune evasion processes. Most of the studies focused on multiple myeloma and chronic lymphocytic leukemia, but in the last decade the putative role of TLRs in other types of B-cell lymphomas has gained much interest. The aim of this review is to discuss recent findings on the role of TLRs in normal B cell functioning and their role in the pathogenesis of B-cell malignancies.

## 

Toll-like receptors (TLRs) are essential receptors of the innate immune system and key regulators of the acquired immune system. Ten proteins (TLR1–10) have been identified in humans [1],[2], each of them with a particular subcellular localization depending on the specific pathogen-associated molecular patterns (PAMPs) or damage-associated molecular patterns (DAMPs) they recognize (Table 1). TLR2 forms functional heterodimers with either TLR1 or TLR6. These heterodimers together with TLR4 and TLR5 are expressed on the cell membrane, whereas TLR3, TLR7, TLR8 and TLR9 are located in endosomes. TLRs induce pro-inflammatory molecules but, they are also implicated in proliferation, survival, and tissue repair [3].

**Table 1:** Toll-like receptors (TLRs) and their DAMPs and PAMPs ligands

	**DAMPS (endogenous)**	**PAMPs (exogenous)**	
	**Ligand**	**Ligand**	**Origin**
TLR1		Tri-acylated Lipopeptides	Bacteria and Mycobacteria
		Soluble factors	*Neisseria meningitidis*
TLR2	HSP60, HSP70, HSP96	Lipoprotein/lipopeptides	Various pathogens
	HMGB1	Peptidoglycan	Gram + bacteria
	Hyaluronic acid	Lipoteichoic acid	Gram + bacteria
		Lipoarabinomannam	*Mycobacteria*
		Phenol-soluble modulin	*Staphylococcus epidermis*
		Glycoinostolphospholipids	*Trypanosoma cruzi*
		Glycolipids	*Treponema maltophilum*
		Porins	*Neisseria*
		Atypical-LPS	*Leptospira interrogans* and *Porphyromonas gingivalis*
		Zymosan	Fungi
TLR3	dsRNA, mRNA	dsRNA	Viruses
TLR4	HSP22, HSP60, HSP70,	LPS	Gram- bacteria
	HSP96	HSP60	*Chlamydia pneumonia*
	HMGB1β-defensin 2	Fusion protein	Respiratory syncytial virus
	fibronectin	Envelope proteins	Mouse mammary tumor virus
	Hyaluronic acid	Taxol	Plant product
	Heparan sulphate		
	Fibrinogen		
	Surfactant-protA		
TLR5		Flagellin	Gram + or Gram- bacteria
TLR6		Di-acylated lipopeptides	Mycoplasma
		Lipoteichoic acid	Gram + bacteria
		Zymosan	*Fungi*
		Phenol-soluble modulin	*Staphylococcus epidermis*
		Heat-liable soluble factor	Group B *streptococcus*
TLR7	Endogenous RNA	ssRNA	Viruses
TLR8	Endogenous RNA	ssRNA	Viruses
TLR9	Endogenous DNA	Unmethylated CpG motifs	Bacteria and viruses
		Hemozoin	Plasmodium
TLR10	Unknown	Unknown	

TLRs are pattern recognition receptors structurally characterized by extracellular leucine rich repeats, and transmembrane and intracellular Toll/Interleukin-1 receptor (TIR) domains [1],[3]. The extracellular domain interacts directly with PAMPs or DAMPs, triggering the downstream signaling through the TIR domain [1]. In mammals, four different types of signaling adaptor proteins can be recruited by the TIR domain: Myeloid differentiation primary-response protein 88 (MyD88), TIR-domain-containing adaptor protein inducing IFNβ (TRIF), TRIF-related adaptor molecule (TRAM), and TIR-domain-containing adaptor protein (TIRAP) (Figure 1). The MyD88 signaling cascade is essential for TLR2, TLR4, TLR5, TLR7, TLR8 and TLR9. TIRAP activation is MyD88-dependent and is associated with TLR2 and TLR4. TRIF acts independently of MyD88 in signal transduction following TLR3 and TLR4 activation. TRAM mediates TLR4 signaling in a MyD88-independent/TRIF-dependent way [1]. The adaptor proteins serve as a scaffold for the recruitment of IL-1R-associated kinases (IRAK) 1, 2, 4 and M and TAB2 and TNF-receptor-associated factor 6 (TRAF6) which eventually leads to nuclear translocation of Nuclear factor kappa-B (NF-kB) [1],[3] (Figure 1). Other transcription factors that can be activated are activator protein 1 (AP-1) and interferon regulatory factor 3 (IRF3) [1].

**Figure 1:**
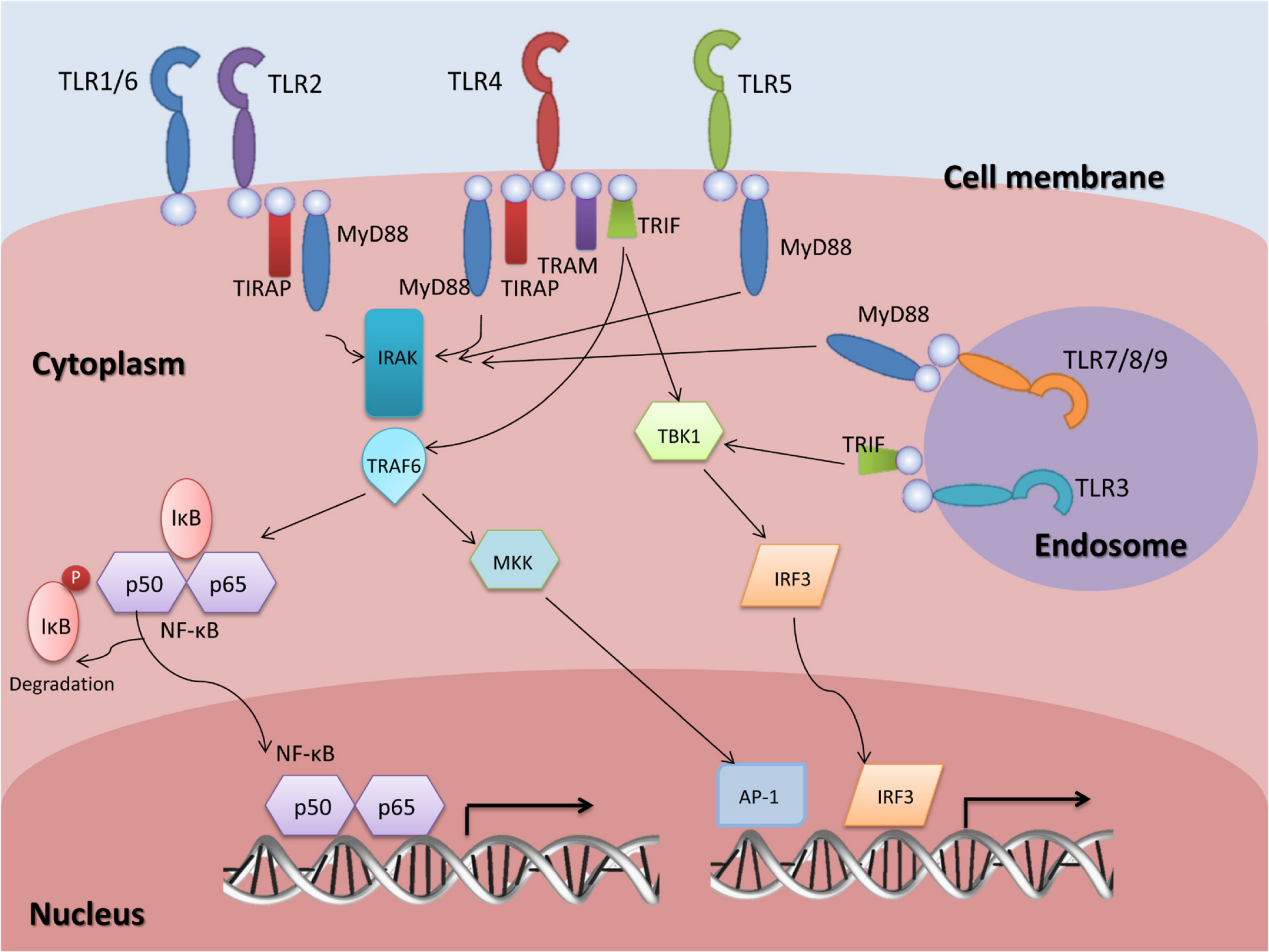
**Signaling pathway of toll-like receptors (TLRs).** After recognition of the ligand by the extracellular domain of TLRs, adaptor proteins are recruited to the TIR domain and initiate intracellular signaling. Four types of adaptor proteins have been identified in mammals: MyD88, TRIF, TRAM, and TIRAP. The recruitment of IRAK and TRAF6 to the adaptor proteins leads to nuclear translocation of NF-kB. TRAF6 can also activate MAPK kinases (MKK) leading to the activation of transcription factor AP-1. This pathway is common to all the TLRs, except for TLR3 which uses TANK-binding kinase 1 (TBK1) and results in the activation of IRF3.

The contribution of TLRs to B-cell lymphoma pathogenesis has gained more interest in recent years [2],[4], but it is not completely understood. This review summarizes the current knowledge on the expression of TLRs in normal B-cells and in B-cell malignancies and discusses how TLRs can contribute to onset and progression of these malignancies.

## Expression and function of TLRs in normal B-cells

Although expression of TLRs in normal B-cells is interesting, what happens in B-cells when they are triggered or the role they play in B-cell maturation and differentiation is more important.

### TLR expression in B-cell subsets

A number of studies investigated expression of TLRs in B-cell subsets isolated from peripheral blood mononuclear cells (PBMC) or tonsil. PBMC derived B-cells express TLR1, TLR6, TLR7, TLR9 and TLR10 [5]. Analysis of peripheral naïve and memory subsets indicates that only TLR1, TLR7 and TLR9 are expressed at low levels in naïve B-cells, while memory B-cells express high levels of TLR6, TLR7, TLR9 and TLR10 [6]. Tonsillar B-cells express less TLR7 than peripheral B-cells, but show higher TLR9 levels [7]. Bone marrow plasma cells express TLR1, TLR8 and TLR9 in a small subset of healthy controls [8].

### Triggering of TLRs in B-cells

Triggering of resting or naïve B-cells through the B-cell receptor (BCR), CD40 or CpG oligodesoxynucleotides (CpG) induces a quick upregulation of TLR7, TLR9 and TLR10 [6],[9]. Triggering of TLR9 on PBMC derived B-cells with CpG induces its downregulation and in addition leads to an upregulation of TLR7 [5]. Bernasconi [6] hypothesized that low or null expression of TLRs in naïve B-cells is a protective mechanism to avoid polyclonal activation. The high and diverse expression of TLRs in memory B-cells on the other hand, might facilitate continuous antibody production. Production of type I interferon (IFN) by plasmacytoid dendritic cells (pDCs) in response to infections positively modulates the expression of TLR7 but not of other TLRs in naïve B-cells [10].

**Figure 2:**
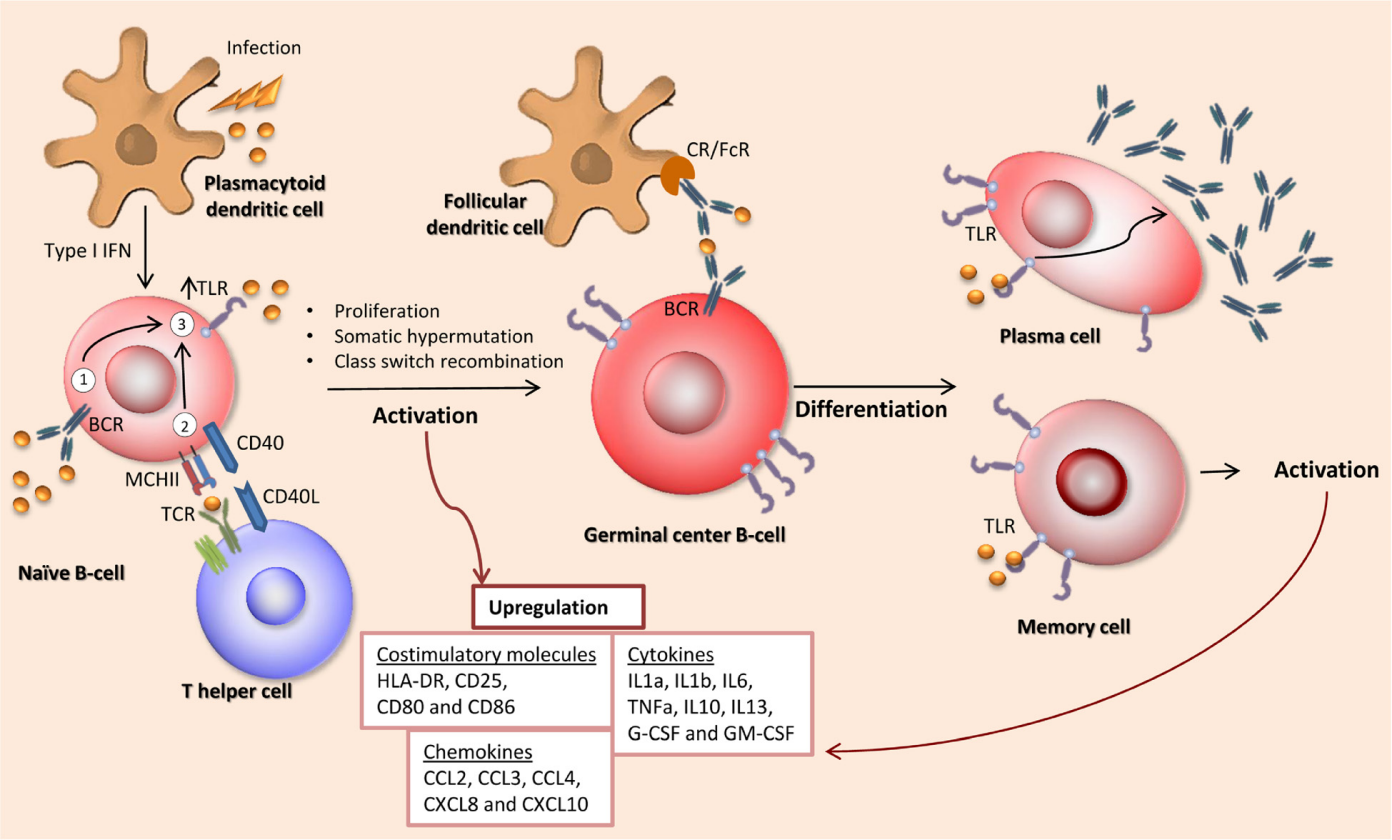
**The role of TLRs in B-cell activation and differentiation.** TLR ligation constitutes one of the three activation signals of naïve-B cells next to BCR triggering and T-cell interaction through CD40. The latter two signals upregulate the expression of TLRs, which subsequently interact with their respective ligands to activate naïve B-cells completely. Activation through TLRs enhances expression of membrane molecules and the production of cytokines and chemokines. It also induces proliferation, somatic hypermutation and class switch recombination. After their first encounter with the ligand and consequent maturation, germinal center cells can further transform into antibody-secreting plasma cells or memory B cells. TLR signaling has been proven to increase antibody secretion and participates in the maintenance of serological memory.

Triggering of TLR1/TLR2, TLR7 and TLR9 in B-cells results in the upregulation of several B-cell activation markers, including HLA-DR, CD25, CD80 and CD86, as well as the production of several cytokines and chemokines [7],[11] (Figure 2). Cytokine production levels were higher in memory B-cells as compared to naïve B-cells, whereas chemokines are produced at similar levels [11].

### The role of TLRs in B-cell maturation and differentiation

Several studies showed that TLRs are involved in the formation of germinal centers (GC) (Figure 2). Stimulation of TLR2+ human B-cells, a small subpopulation found in blood or spleen, causes a GC like differentiation with upregulation of CD77 and production of CXCL13, CXCL8 and IL-6 [12]. In TLR7 deficient mice no GC B-cells are formed after immunization with Friend Virus [13], whereas in IL-21R^ko^ mice, GC formation is dependent on the presence of TLR7 ligands [14].

Several studies demonstrate a crucial role of TLR triggering in the induction of class switch recombination (CSR). Stimulation of TLR4 with LPS can induce IgG2b CSR, while addition of IL-4 blocks this process [15]. CpG stimulation of TLR9 in the presence of MYD88, induces CSR to IgG2a, IgG2b and IgG3 in murine B-cells [16]. In human naïve (CD19+ CD27- IgG/A- ABCB1+) B-cells, TLR9 stimulation with CpG was required for CSR to IgG and IgA, in addition to BCR ligation and T-cell help [17]. T-cell independent stimulation of TLR9 with CpG in combination with IL-10 treatment of naïve B-cells enhances expression of activation induced deaminase (AID) and the subsequent CSR to IgG1, IgG2, and IgG3 [18]. Upper respiratory mucosal B-cells can be stimulated with dsRNA via TLR3 and TRIF, to activate NF-κB and induce expression of AID and CSR to IgG and IgA [19]. BCR independent activation of murine resting B-cells via TLR2 and CD40 causes proliferation and differentiation to marginal zone precursor cells that have upregulated AID expression [20].

Somatically mutated memory B-cells can be generated from immature transitional B-cells upon CpG treatment. Most of the somatic hypermutations were observed in B cells containing VH1, VH4 and VH6 gene segments [21]. This study indicates that somatic hypermutation is dependent on or can be induced by TLR triggering.

Differentiation to plasma cells and the production of antibodies are two processes in which TLRs also play an important role (Figure 2). Several immunization studies in mice show that TLRs are essential for the production of antibodies. T-cell dependent immunizations in MyD88 KO mice do not lead to the production of antibodies, while triggering of TLR4 during the immunization procedure of wild type mice induces a significant increase in antibody titers [22]. Stimulation of TLR4 and TLR7 significantly enhanced antibody titers upon immunization [23]. Immunization with CpG linked to the protein antigen enhances B-cell proliferation and plasma cell differentiation [24]. For induction of an anti-viral response, MYD88 expression in B-cells was essential [13],[25]. Naïve B-cells stimulated by CpG start proliferating and differentiate to IgM producing plasma cells with increased surface expression of molecules involved in antigen presentation such as HLA-DR, CD40 and CD80. These cells indeed have an increased potential to activate allogeneic T-cells [26]. Cord blood transitional B-cells express TLR9 and respond to CpG by expression of AID and BLIMP-1 and produce anti-pneumococcal antibodies as a first line defense at birth [27]. CpG stimulation of TLR9 positive IgM memory B cells drives differentiation to plasma cells that produce IgM antibodies to Streptococcus pneumoniae [27].

In summary, multiple studies show participation of TLRs in adaptive immunity, including modulation of the activation of B- and T-cells. In B-cells TLRs have been proposed to constitute the third essential signal for complete activation [17], along with BCR triggering and interaction with T-cells. Additionally, it has been shown that TLRs are involved in B-cell maturation by CSR, somatic hypermutation, induction and maintenance of GC and B-cell memory, differentiation to plasma cells and the production of antibodies. Considering the amount of evidence supporting TLR-MyD88 involvement in acquired immune responses mediated by B-cells, it is likely that they are involved in the pathogenesis of B-cell malignancies.

## TLR expression in B-cell malignancies

B-cell malignancies are classified based on the resemblance of the tumor cells to specific maturation stages of normal B-cells, e.g. the normal counter part of follicular lymphoma (FL), Burkitt lymphoma (BL) and diffuse large B-cell lymphoma (DLBCL) are related to germinal center B-cells, while multiple myeloma (MM) is a plasma cell malignancy.

### Expression in multiple myeloma

TLR1, TLR2, TLR3, TLR4, TLR7, TLR8, and TLR9 have been reported to be expressed in MM derived cell lines. Of these TLR1, TLR4, TLR7, and TLR9 are expressed most commonly [8],[28]. Analysis of tumor cells from sorted bone marrow mononuclear cells of MM patients showed high TLR2, TLR4 and TLR9 mRNA levels and these findings were consistent with the high protein expression of TLR4 and TLR9 when analyzed by flowcytometry [29]. Overall, the expression of TLR4 and TLR9 is consistently reported in MM cell lines or primary cells.

### Expression in chronic lymphocytic leukemia (CLL)

TLR1, TLR2, TLR6, TLR7, TLR9 and TLR10 were expressed in CLL, while the other TLRs were low or negative [30]-[32].

### Expression in other B-cell malignancies

For other B-cell lymphoma types, studies on TLR expression are limited. In acute lymphoblastic leukemia (ALL) cell lines, TLR1, TLR2, TLR3, TLR4, TLR6 and TLR7 are expressed albeit at variable levels [33]. In bone marrow of ALL patients with >90% blasts TLR2 mRNA can be detected in the majority of the samples [33]. BL cell lines express TLR7 and TLR9 [34]. Mucosa associated lymphoid tissue (MALT) lymphoma show strong expression of TLR4 and weak expression of TLR5 [35]. In mantle cell lymphoma (MCL), TLR1, TLR4, TLR7, TLR9 and TLR10 exhibit significant mRNA levels, whereas TLR2, TLR3, TLR5 and TLR8 are negative [36]. A significant expression of TLR2 and TLR8 at both the protein and mRNA level was found in DLBCL [37]. In comparison to reactive lymph node, TLR2 levels were high in DLBCL and TLR5 low in follicular lymphoma (FL). TLR3, TLR6, TLR7 and TLR9 expression levels were similar in DLBCL, FL and peripheral T cell lymphoma [37].

Polymorphisms in TLRs have been shown to increase the risk of non-Hodgkin lymphoma [38], as well as in Hodgkin lymphoma [39]-[41].

Thus, it is evident that B-cell malignancies display a wide range of TLR expression patterns. Nevertheless, it remains to be elucidated if their expression resembles a normal B-cell phenotype or if it is a consequence of the malignant transformation.

## Potential role of TLRs in B-cell malignancies

**Figure 3:**
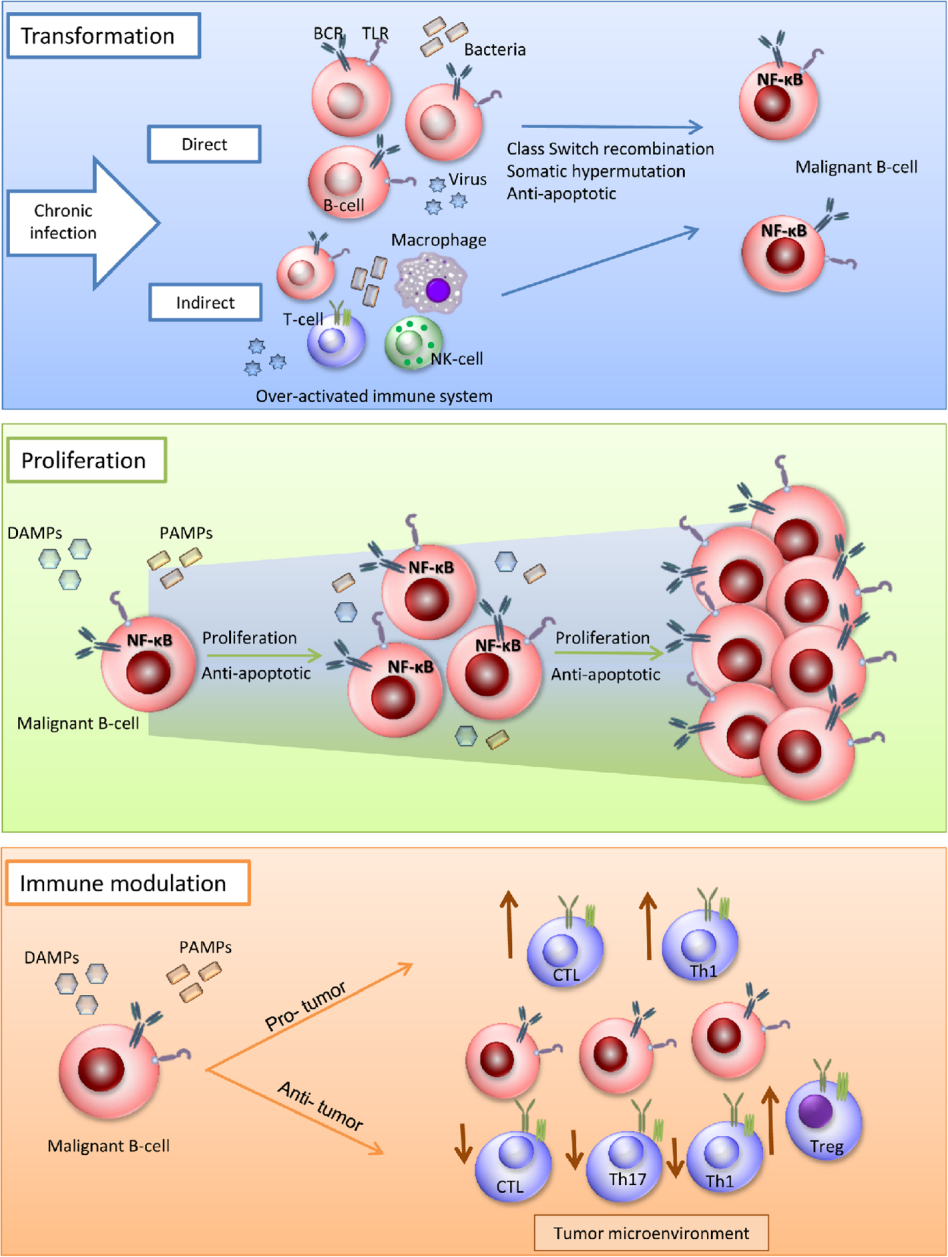
**Possible roles of TLRs in the pathogenesis of human B-cell lymphomas.** There are three possible mechanisms by which TLRs favor the pathogenesis of B cell malignancies. First, they can indirectly induce B-cell transformation by increasing the probability of mutations and double stranded DNA breaks after activation of naive B-cells or by favoring a pro-inflammatory microenvironment that increases the chance of mutations. Secondly, TLRs can direct the selection of a malignant clone by increasing proliferation and inhibiting apoptosis through the specific activation of NF-κB. Finally, malignant B-cell can modulate the immune response through stimulation of TLRs in two ways possibly depending on the cell type: The-pro tumor response favoring the increase of suppressor cells (like Tregs), and the decrease of a cytotoxic response (by downregulating Th1, Th17 and CTL) or an anti-tumor response by inreasing immunogenicity and increasing the sensitivity to CTLs and upregulating Th1.

The survival and proliferative mechanisms used by malignant cells usually includes aberrant activation of signaling and regulatory pathways that are also used in their normal counterparts. Three possible pathogenic mechanisms can be anticipated for the TLRs which will be discussed below (See Figure 3).

### Role of TLRs in the transformation of normal B-cells into malignant B-cells

One of the main indications that TLR are involved in the pathogenesis of B-cell malignancies comes from studies focusing on its adaptor protein MyD88. Knockdown of MyD88 in activated B cell type (ABC) DLBCL cell lines causes a marked decrease in proliferation, this effect was shown to be dependent on the presence of oncogenic mutations [42]. Specific oncogenic mutations in the Toll/IL-1 receptor (TIR) domain of MyD88 are found in 91% of lymphoplasmacytic lymphomas [43], 29% of ABC DLBCL, 9% of gastric MALT lymphomas [42], 13% of splenic marginal zone lymphomas (SMZL) [44], 2.9% of CLL cases and 5.6% of IgH-mutated CLL cases [45].

NF-κB is constitutively activated in various lymphoma subtypes and presents an important pathway to evade cell death. This is demonstrated by the frequent occurrence of somatic mutations in NF-κB related genes in mature B-cell lymphomas [46]. As NF-κB can be activated via triggering of the TLRs, this might provide a potential mechanism to avoid apoptosis and increase their proliferation rates. Consistent with this, triggering of TLR4 and TLR9 in MM resulted in nuclear translocation of NF-kB, and enhanced cell growth and IL-6 production [29]. In a similar way, ligation of TLR1/2 and TLR6/2 increased tumor cell survival in CLL by triggering NF-κB signaling [32].

Several B-cell lymphomas have been associated with viral or bacterial infections [47]-[50]. This led to the speculation that acute and sustained infections trigger malignant B-cell transformation via activation and induction of a prolonged proliferative state, favoring accumulation of genetic aberrations. The strongest evidence supporting this hypothesis is the regression and prolonged remission of gastric MALT lymphoma after treatment of *Helicobacter (H.) pylori* infections [51]-[53]. Interestingly, there is a correlation between infection and TLR expression pattern reported for specific lymphoma subtypes. MALT lymphomas express TLR4 [35] which recognizes LPS derived from Gram-negative bacteria like *H. pylori*[48]. BL and DLBCL express TLR7, TLR8 and/or TLR9 [34],[37], which mainly recognize single-stranded RNA or unmethylated CpG motifs from viruses. These lymphoma subtypes, although for DLBCL in only a small subpopulation of patients, have been associated with viruses like Epstein-Barr, Human immunodeficiency virus and HCV [47],[49],[50]. As TLRs have been shown to affect somatic hypermutation and class switch recombination [15],[18],[19],[21],[27] it can be speculated that TLR triggering also facilitates non-Ig somatic aberrations that leads or contributes to the malignant transformation of germinal center B-cells.

In summary, the above mentioned studies provide evidence, although still mostly speculative and future analyses are required, for a possible role of TLRs in the malignant transformation via continuous TLR triggering following chronic infections or via induction of a cellular conditions that is prone to somatic mutations or DNA breaks.

### TLRs in proliferation of malignant B-cells and lymphoma progression

Several studies showed induction of proliferation of malignant B-cells upon TLR triggering. Stimulation of TLR9 by CpG in 31 B-cell lymphoma samples strongly induced proliferation in CLL, revealed intermediate effects in MZL, CLL/SLL, FL and DLBCL and no effect in MCL and ALL [30],[33]. ALL cells also did not respond to stimulation of TLR2 and TLR7 [33]. TLR4 triggering in MCL did show enhanced proliferation [36]. In CLL stimulation of TLR9 revealed more proliferation in unmutated compared to mutated IgH cases. Mutated cases showed a G1/S cell cycle arrest and increased number of apoptotic cells [54]. In MM cells, ligands for TLR1, TLR2, TLR4, TLR5, TLR6, TLR7 and TLR8 enhanced IL-6 dependent proliferation [8],[28]. Activation of TLR7 and TLR9 protected MM cells from serum-deprivation or dexamethasone induced apoptosis [28]. One of the main contributors to the enhanced proliferation is probably achieved via the induction of NF-κB, which also protects the cells from apoptosis (see above).

Together, these findings indicate that aberrant TLR activation in B-cell lymphoma may contribute to their proliferative potential and protect cells from apoptosis.

### TLRs in immune modulation

TLRs have been proposed to contribute to the pathogenesis of B-cell malignancy as modulators of the immune system. Putative mechanisms involve modulation of pro- and anti- inflammatory cytokines and costimulatory molecules, induction of regulatory cells and alteration of T-cell function.

Triggering of TLR7 and/or TLR9 enhanced expression of co-stimulatory molecules, cytokines and/or chemokines in BL, CLL and ALL [30],[31],[34],[55],[56]. This indicates that the tumor cells become more immunogenic. Indeed, activation of TLR7 increases the sensitivity of CLL cells to cytotoxic T-cells [31],[57]. LPS pre-treated primary MCL cells are able to resist killing by allogeneic cytotoxic T-cells by producing IL-10 and VEGF [36]. Triggering of ALL cells with the TLR9-ligand, failed to promote T-cell proliferation by primary ALL cells, but turned the T-cell cytokine profile towards Th1 by upregulating IFN-γ production of T-cells [57]. TLR2-stimulated ALL cell lines also enhanced expression of co-stimulatory markers and IFN-γ, but failed to induce T-cell proliferation [33]. In the normal B-cell response, enhancement of IL-6, IL-10, or IFN-γ in response to TLR stimulation has been correlated to immunosuppressive functions. IL-10 production by B-cells after TLR4 or TLR9 triggering suppresses Th1 and Th17 cells [58] and after TLR2 stimulation induces IL-10 producing T regulatory (Treg) cells [59]. Patients with MM or lymphomas, such as FL, DLBCL and primary mediastinal B-cell lymphoma, frequently have high numbers of Treg cells in the microenvironment [60],[61]. In addition, the *in vivo* and *in vitro* generation of Treg cells by B-cells was recently reported to be MyD88-dependent indicating another link with TLR stimulation [59].

Overall, there is clear evidence that supports a putative role of the TLRs in the modulation of the immune response and microenvironment in B-cell malignancies. The presence and activation of TLRs induces different mechanisms depending on the lymphoma subtype, the stimulated TLR and the microenvironment. TLR stimulation can favor proliferation of malignant B-cells by facilitating immune evasion through Treg induction and production of immunosuppressive cytokines. In contrast, in some situations, TLRs can stimulate resolution of the tumor by encouraging a cellular-mediated immune response.

## Therapeutic perspectives

A main question to be addressed is: what are the effects of TLR agonists *in vivo*? In a mouse model for ALL, treatment with CpG oligonucleotides gave long term protection from ALL, by inducing a Th1 response [62],[63]. Topical administration of Imiquimod, the ligand for TLR7, resolves skin manifestations in CLL patients and increased expression of co-stimulatory molecules on leukemic tumor cells [64]. A phase I study of TLR9 stimulation combined with rituximab in non-Hodgkin lymphoma showed no toxicity, induction of interferon and interferon inducible genes and an overall response rate of 32% (6/19) [65]. The phase II follow up study in relapsed and refractory FL patients, revealed enhanced antibody-dependent cell-mediated cytotoxicity in 11/23 patients, and 74% of patients were alive without progressive disease at day 90 [66]. At least six clinical trials evaluating agonists for TLR3 (1 Trial), TLR7/8 (2 Trials) and TLR9 (3 Trials) in B non-Hodgkin lymphomas were in progress at January of 2008 [67]. Most of these have been terminated for different reasons, or the results have not been published yet. A phase I/II trial in CLL with a TLR7 agonist showed that part of the patients could be sensitized for vincristine [68], as had also been shown *in vitro*[69]. Notably, the effectiveness of several TLR agonists has been reported to be low in Phase III studies, so the number of research groups following this direction has decreased [67].

## Conclusion

TLRs have been suggested as promoters of malignant transformation, tumor cell maintenance and progression in B-cell malignancies. The TLR expression patterns are diverse, not completely known yet for each B-cell malignancy, and could be normal for the B-cell phenotype or a consequence of transformation. TLR stimulation induced different effects in B-cell malignancies due to specific aberrations in the tumor cells or by differences in the tumor microenvironment. Despite these uncertainties, it is very likely that TLRs participate in the development and survival of malignant B cells.

There is a strong correlation between chronic infections and the development of some specific types of B-cell lymphoma. In these subtypes, it is likely that TLRs are directly involved in malignant transformation. In other B-cell malignancies, such as MM and CLL, TLRs appear to participate in immune evasion and tumor progression. It is evident that extreme precaution should be taken when considering the use of TLR agonists as (adjuvant) therapy in B-cell malignancies, because these agonists may have tumor-promoting properties.

## Competing interests

The authors have no competing interests to disclose.

## Authors’ contributions

JIC, ZL and LV contributed to the literature analysis/interpretation and manuscript writing. AD, AvdB and LV edited/revised all drafts. All Authors approved the final version of the manuscript.

## Authors’ information

JIC is a PhD student working on innate immunity. ZL is a PhD student, ENT physician and oncologist. AvdB is a molecular biologist working in lymphoma and non-coding RNA research. AD is a pathologist/scientist working in lymphoma and EBV-related research. LV is a scientist working in the field of lymphoma and tumor immunology.
